# Impact of early nutrition on brain development and neurocognitive outcomes in very preterm infants

**DOI:** 10.1038/s41390-025-03964-8

**Published:** 2025-03-04

**Authors:** Nima Naseh, Tânia F. Vaz, Hugo Ferreira, Nuno Canto Moreira, Lena Hellström-Westas, Fredrik Ahlsson, Johan Ågren

**Affiliations:** 1 Department of Women’s and Children’s Health, Uppsala University, Uppsala, Sweden; 2 Institute of Biophysics and Biomedical Engineering, Faculty of Sciences, University of Lisbon, Lisbon, Portugal; 3 Departments of Neuroradiology, Uppsala University Hospital and Karolinska University Hospital, Uppsala, Sweden

## Abstract

**Background:**

Malnutrition of preterm infants may negatively affect brain growth and later neurocognitive function. We aimed to investigate the association between very preterm infants’ macronutrient intakes, and brain MRI at term and neurodevelopment at 2 years.

**Methods:**

Single-center, retrospective cohort including extremely (22–27w) and very (28–31w) preterm infants born 2011–2014. The intakes of fluid, protein, carbohydrate, fat, and total calories during days 0–28 together with body weights were assessed in relation to brain MRI (morphology, volumetry, diffusion-weighted imaging) at term, and cognition (BSID-III) at 2 years, using adjusted multivariable regression analyses.

**Results:**

Seventy-two infants were included. A lower (*p* < 0.001) caloric intake in extremely preterm (*n* = 26) than in very preterm (*n* = 46) infants did not translate to any differences in brain volumes. While bivariate correlations (*p* < 0.01) were found between the enteral intakes of all macronutrients, and white matter volume and apparent diffusion coefficients, none of the correlations remained significant after adjusting for covariates in the multivariable analysis. Similarly, no associations between nutrient intakes and cognitive development remained after covariate adjustment.

**Conclusion:**

In a cohort of preterm infants receiving macronutrient intakes meeting current recommendations, individual variations in nutrition did not influence brain growth or neurodevelopment.

**Impact:**

- Early postnatal macronutrient intake was not associated with brain volumes at term or neurocognitive outcomes at 2 years in very preterm infants
- All infants received nutritional intakes meeting current recommendations
- Adequate macronutrient intake based on a standardized protocol may eliminate the need for further minor adjustments in the pursuit of supporting brain growth and neurodevelopment in preterm infants.

## Background

A highly important long-term health outcome of early life experiences in infants born preterm is neurodevelopment which, to a large extent, is pre-programmed and thus experience-independent. There are however several important and at least partly modifiable environmental factors that might impact brain growth and differentiation, such as infection/inflammation, oxidative stress, environmental enrichment, and nutrition. Of these, the latter is directed almost entirely by medical caregivers and can from a simplistic view either optimally support neurodevelopment with normal fetal growth serving as the reference or be left ignored and confer an additional risk of central nervous system harm. The late stages of gestation are critical for brain growth and development, and preterm neonates are particularly vulnerable to disruptions in this process since a larger proportion of their neurogenesis and maturation occur outside the womb.^1^

Despite efforts to enrich enteral feeds and, to optimize supportive parenteral nutrition, it has been demonstrated that preterm infants later have smaller brain volumes than term-born infants, and this is paralleled by impairment of neurocognitive development.^2^ Infants born extremely preterm, those with a gestational age of less than 28 weeks, are at the highest risk of postnatal malnutrition due to enteral feeding intolerance, deficient enteral uptake, and limited nutrient reserves.^3^ While studies in developmental neuropathology emphasize the important role of nutrition in shaping brain structure, the specific impact of macronutrients on brain volume remains unclear.^4–7^

The present study aimed to investigate the impact of nutritional provision on brain development in preterm infants, based on the hypothesis that the amount, route and/or composition of nutrients provided during the first weeks may influence brain volumes and later cognition.

## Methods

### Subjects

**Fig. 1:**
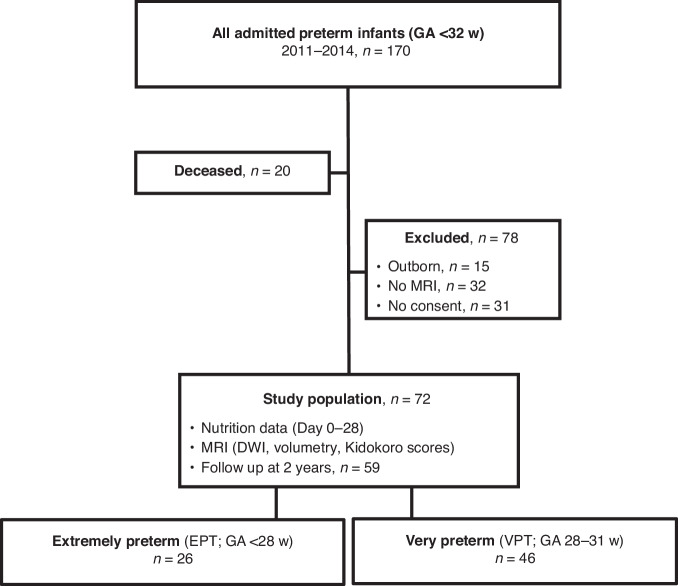
STROBE flowchart of the study cohort.

All very preterm (VPT, *n* = 170) infants with a gestational age (GA) of less than 32 weeks cared for at Uppsala University Children’s Hospital from July 2011 to December 2014 were considered eligible for participation (Fig. 1). Among the 170 infants, 150 survived and 118 had brain magnetic resonance imaging (MRI) performed at term as part of the clinical routine. Informed parental consent for study inclusion was obtained for 87 infants and after exclusion of 15 outborn infants, for whom full nutritional data could not be retrieved, 72 infants remained to constitute the final study cohort (Fig. 1).

### Perinatal characteristics

Data collected included gestational age (GA) (in days, estimated using routine second trimester ultrasound), antenatal steroid exposure and timing of administration, mode of delivery, birth weight (z-score), Apgar scores, and sex (Table 1). The number of days on mechanical ventilation was used as a proxy for illness severity. Bronchopulmonary dysplasia (BPD) was defined as the receipt of supplemental oxygen at 36 weeks postmenstrual age (PMA).^8^ Sepsis was defined by clinical symptoms and either a positive blood culture or clearly (>25 mg/dL) elevated C-reactive protein. Necrotizing enterocolitis (NEC) was defined as a histopathological diagnosis after laparotomy. Persistent ductus arteriosus (PDA) treatment included surgical ligation or catheter-based closure. Retinopathy of prematurity (ROP) was classified and staged according to The International Classification of Retinopathy of Prematurity^9^ with treatment being either laser photocoagulation or intravitreal injection of anti-VEGF.^10^

**Table 1:** Clinical characteristics and neurodevelopmental assessment of study cohort.

	All infants, GA < 32w (*n* = 72)	VPT infants, GA 28-31w (*n* = 46)	EPT infants, GA < 28w (*n* = 26)
Antenatal steroids	65 (90)	39 (85)	26 (100)
Gestational age (weeks)	28.1 ± 2.6	29.7 ± 1.2	25.1 ± 1.4*
Male	33 (46)	23 (50)	10 (38)
Cesarean section	46 (64)	33 (72)	13 (50)
5 min Apgar	7.7 ± 1.2	8.6 ± 1.2	7.2 ± 1.4
Birth weight (g)	1190 ± 401	1401 ± 328	824 ± 201*
Birth weight z-score	−0.98 ± 1.2	−1.29 ± 1.4	−0.44 ± 0.7*
Mechanical ventilation (days)	8.6 (0–82)	0 (0–10)	11 (0–82)*
Sepsis	21 (29.2)	7 (15.2)	14 (54)*
Necrotising enterocolitis surgery	1 (1)	0	1 (4)
Persistent ductus arteriosus ligation	6 (8)	0	6 (23)*
ROP treatment	1 (1)	0	1 (4)
BSID-III cognitive index^a^	103 ± 14	103 ± 15	102 ± 14
BSID-III motor index^a^	101 ± 12	103 ± 13	99 ± 11
BSID-III language index^a^	101 ± 17	100 ± 16	102 ± 18
Neurodevelopmental impairment	11 (19)	4 (11)	7 (29)

Data are *n* (%), mean ± SD, or median (range).

*GA* Gestational age, *VPT* very preterm, *EPT* extremely preterm, *ROP* retinopathy of prematurity, *BSID-III* Bayley Scales of Infant Development 3rd Ed.

*p < 0.05 in VPT vs EPT.

^a^Means calculated to account for missing data in individual indices.

### Nutrition data

All nutrients provided were retrieved from electronic medical records and nutrition charts and entered into the macro- and micronutrient calculation software Nutrium™ (Nutrium AB, Umeå, Sweden), to determine the cumulative intake per kilogram body weight of fluid, calories and macronutrients (protein, carbohydrate, lipid) for the first seven, and 28 days. Changes in body weight were calculated as z-scores and percentages.

The institutional nutritional protocol used during the study period was based on current ESPGHAN recommendations.^11^ In brief, a volume-based protocol, adjusted for weight and adapted to GA was used with a starting total fluid intake ranging from 120 (at 22 weeks GA) to 80 (at 27 weeks GA) mL/kg/day, subsequently adjusted to limit a maximal initial weight loss to 10–15%, and to regain birth weight by 7–14 days. All very preterm infants received either mother’s own milk or donor breast milk, with feeds started within 2 h after birth and continuing every two hours, and with the remaining nutritional needs met by parenteral nutrition. Daily increments of enteral feeds were 20–40 mL/kg/d to a target of 150–170 mL/kg/day within 1-2 weeks depending on gestational age at birth and enteral tolerance.

According to our institutional protocol all infants exclusively receive human (mother’s own, or donor) milk, and fortification is initiated at a postnatal age of four days, or after removal of the umbilical artery catheter in which a sodium-free isotonic amino-acid solution is infused at a rate of 0.5 mL/h in order to keep the line open. The target protein content is 2.4 g/100 mL until a PMA of 28 weeks is reached (4 g/kg/d at 170 ml/kg/d; protein-to-energy ratio ~3.2 g/100 kcal), and 2.1 g/100 mL thereafter (3.5 g/kg/d at 170 ml/kg/d; protein-to-energy ratio ~3.0 g/100 kcal), using PreNAN FM 85 (Nestlé AB, Helsingborg, Sweden). Breast milk is analyzed every two weeks by the Children’s Hospital Nutrition Unit, using 10 ml samples in a breast milk analyzer (Miris, Uppsala, Sweden) after pooling of maternal milk collected over 24 h. Similarly, each batch of donor milk is tested and fortified accordingly.

### Brain MRI analysis

The MRIs were performed at term PMA 40.0 ± 1.7 (SD) weeks using 1.5 T scanner (Siemens Avanto, Germany) and an imaging protocol including T2-weighted Fast Spin Echo, T2*-weighted Gradient Echo, Volumetric T1-weighted, and Diffusion-Weighted Imaging (DWI; Single-shot EPI sequences, b-value = 0 and 800 s/mm²). Apparent diffusion coefficients (ADC) were, as previously described,^12,13^ derived from the DWI data measured in the left periventricular white matter, left basal ganglia, and the pons, by outlining circle-shaped regions-of-interest with predefined radii (Supplementary Fig. S1). The assessments were made by two assessors (N.N. and N.C-M.), blinded to the individual infants’ clinical characteristics and outcomes. While a formal intraclass correlation coefficient analysis was not performed, any discrepancies between the assessors were evaluated by comparing them with the original neuroradiologist’s report to ensure accuracy. Anatomical assessments of both supra- and infratentorial brain structures included intraventricular hemorrhage (IVH) graded according to Papile,^14^ white matter lesions, ventricular dilatation, basal ganglia injury, and cerebellar hemorrhage. MRIs were also scored according to Kidokoro.^15^

Brain volume analysis utilized a semi-automated segmentation technique known as Morphologically Adaptive Neonatal Tissue Segmentation (MANTiS) to estimate total brain volume, white matter volume, cortical gray matter volume and cerebellar volume (Supplementary Fig. S2).^16,17^

### Neurodevelopmental assessment

Infants were assessed according to a standardized follow-up program including motor and cognitive assessment at pre-specified ages. Formal testing was made by a child psychologist using the Bayley Scales of Infant and Toddler Development, 3rd edition^18^ (BSID III), at 2 years of age. A score below 85 in at least one BSID domain (cognitive, language, motor) and/or a diagnosis of cerebral palsy was categorized as neurodevelopmental impairment (NDI).

### Statistical analysis

Descriptive parametric and non-parametric tests were utilized for comparisons (mean, standard deviation, median, range), as applicable. Analyses was conducted for the entire group and then separately for two subgroups, extremely preterm (EPT, GA < 28 weeks) and VPT (GA 28-31 weeks), and further stratified by sex. Statistical significance was determined using Fisher’s exact test or the Mann–Whitney U test. Bivariate Pearson and Spearman correlation (depending on the distribution of data) and regressions were used to evaluate the associations between the exposures a) cumulative nutrition provided (Table 2), and b) hospital morbidities, and the outcome variables a) MRI-analyses, and b) neurodevelopment at 2 years.

**Table 2:** Weight change, feeding progression, and nutritional intakes.

	All infants, GA < 32w (*n* = 72)	VPT infants, GA 28-31w (*n* = 46)	EPT infants, GA < 28w (*n* = 26)
Maximum weight loss (%)	9 ± 5	8 ± 3	12 ± 7
Exceeded birth weight			
Day 7	20 (28)	9 (20)	11 (42)*
Day 14	56 (78)	33 (72)	23 (88)
Proportion enteral feeds of total day 7	51 (71)	39 (84)	12 (47)*
Enteral feeds only by day 14	48 (67)	38 (83)	10 (38)*
Total fluid intake (mL/kg/day)			
Day 0–28	169 ± 45	175 ± 17	163 ± 7*
1^st^ week	128 ± 21	129 ± 24	125 ± 14
Caloric intake (kcal/kg/day)			
Day 0–28	131 ± 14	137 ± 15	125 ± 9*
1^st^ week	88 ± 16	90 ± 18	84 ± 8
Protein intake (g/kg/day)			
Day 0–28	3.7 ± 0.4	3.8 ± 0.5	3.7 ± 0.4
1^st^ week	2.4 ± 0.6	2.3 ± 0.6	2.6 ± 0.4
Fat intake (g/kg/day)			
Day 0–28	6.3 ± 1.0	6.8 ± 1.1	5.9 ± 0.7
1^st^ week	4.1 ± 1.1	4.4 ± 1.1	3.5 ± 0.5
Carbohydrate intake (g/kg/day)			
Day 0–28	13.8 ± 1.8	14.2 ± 2.3	13.4 ± 0.9
1^st^ week	10.2 ± 1.9	10.2 ± 2.2	10.3 ± 1.2

Data are n (%), or mean ± SD.

*p < 0.05 in VPT vs EPT.

The associations between nutrition data and MRI assessments were analyzed using multivariable and logistic regression models, as applicable, and were adjusted for gestational age, days on mechanical ventilation, and postmenstrual age at the time of the MRI examination. Since tests of collinearity demonstrated that gestational age and birth weight had notably similar associations, birth weight z-scores were chosen alongside the other covariates in the analysis. Similarly, the association between nutrient intakes and BSID III scores were analyzed in a multivariable regression analysis adjusted for the above covariates (except age at MRI). The same covariates were included in the evaluation of the correlation between NDI and nutrition data, using a binary logistic regression analysis. Data were analyzed using SPSS Statistical software (Version 29 for Windows, SPSS Inc. Chicago, IL) with *p*-values < 0.05 considered statistically significant.

## Results

The demographic and clinical characteristics of the cohort are presented in Table 1. No infant received postnatal corticosteroids, or insulin.

### Nutrition

Table 2 displays the proportion of enteral intake as well as the cumulative total parenteral and enteral intakes during the first four weeks after birth. Mean daily fluid intake and 28 days cumulative caloric intake were higher in the VPT than in the EPT group, with statistical significance determined using a Mann–Whitney U test (*p* < 0.001).

### MRI

The MRI assessments are presented in Table 3. No significant differences in brain volumes, or Kidokoro scores (data not shown), were observed between the EPT and VPT subgroups.

**Table 3:** Brain MRI morphology, volumetry and diffusion‐weighted imaging.

	All infants, GA < 32 w (*n* = 69)	VPT infants, GA 28-31 w (*n* = 43)	EPT infants, GA < 28 w (*n* = 26)
Postmenstrual age at MRI, weeks	40 (1.7)	39.7 (1.4)	40.6 (2)
No IVH	49 (71)	31 (67)	18 (69)
IVH grades 1-2	23 (33)	12 (33)	8 (31)
IVH grades 3-4	0	0	0
White matter injury	9 (13)	7 (15)	2 (8)
Volumetry			
Total brain, mL	377 (45)	382 (47)	368 (41)
Cerebellum, mL	26 (4)	26 (3)	26 (5)
White matter, mL	113 (23)	121 (22)	101 (20)*
Apparent Diffusion Coefficients			
White matter, 10^−6^ mm^2^/s	1423 (159)	1432 (168)	1507 (142)
Pons, 10^−6^ mm^2^/s	987 (132)	971 (152)	1016 (76)
Basal ganglia, 10^−6^ mm^2^/s	1077 (62)	1071 (59)	1087 (67)

Data are mean ± SD, or *n* (%).

*GA* Gestational age, *VPT* Very preterm, *EPT* Extremely preterm, *IVH* Intraventricular hemorrhage.

*p < 0.05 in VPT vs EPT.

### Neurodevelopmental assessment

Follow-up data at 2 years corrected age were available for 59/72 (82%) infants, 49/72 (68%) had BSID III scores, while 10/72 (14%) had been assessed by a psychologist through child habilitation services (Table 1). Eleven infants were categorized as having NDI, and although they mainly belonged to the EPT group the difference between the subgroups was not statistically significant.

### Associations between perinatal data, nutrition, MRI, and neurodevelopment

For the whole cohort the bivariate correlation (Spearman) yielded significant positive associations between white matter volume and 28 days cumulative fluid-intake (*p* = 0.002, r = 0.440), fat (*p* = 0.002, r = 0.405), carbohydrates (*p* = 0.001, r = 0.429), protein (*p* = 0.001, r = 0.425), and calories (*p* = 0.004, r = 0.421). White matter ADC in the VPT subgroup correlated (Spearman) with enteral fluid intake (*p* = 0.002, r = 0.610), fat (*p* < 0.001, r = 0.647), carbohydrates (*p* = 0.005, r = 0.562), protein (*p* = 0.003, r = 0.585), and calories (*p* = 0.002, r = 0.611). However, none of these correlations remained significant after adjusting for covariates in the multivariable regression (Table 4).

**Table 4:** Multivariable linear regression of 28 days nutritional intakes versus brain volumes, apparent diffusion coefficients, and neurodevelopment.

	Fluid		Calories		Protein		Fat		Carbohydrate	
	B (95% CI)	*p*	B (95% CI)	*p*	B (95% CI)	*p*	B (95% CI)	*p*	B (95% CI)	*p*
White matter	0.08 (−0.07; 0.23)	0.284	0.11 (−0.03; 0.26)	0.122	2.92 (−2.26; 8.10)	0.261	0.77 (−1.31; 2.86)	0.459	0.81 (−0.17; 1.78)	0.104
Total brain	−0.14 (−0.39; 0.12)	0.286	0.01 (−0.25; 0.26)	0.969	1.97 (−6.84; 10.79)	0.653	0.69 (−2.83; 4.21)	0.693	−0.37 (−2.06; 1.33)	0.663
Cortical gray matter	−0.20 (−0.45; 0.05)	0.113	−0.11 (−0.36; 0.13)	0.358	−1.01 (−9.88; 7.86)	0.819	−0.28 (−3.81; 3.26)	0.875	−1.12 (−2.79; 0.55)	0.183
ADC White matter	−0.05 (−1.00; 0.89)	0.914	0.42 (−0.47; 1.31)	0.344	11.42 (−17.56; 40.41)	0.430	7.28 (−4.75; 19.32)	0.228	−0.75 (−6.98; 5.48)	0.809
ADC Basal ganglia	0.03 (−0.45; 0.50)	0.910	0.02 (−0.43; 0.47)	0.933	6.30 (−8.29; 20.89)	0.387	1.25 (−4.93; 7.42)	0.685	−0.88 (−4.01; 2.25)	0.572
Cognitive score	−0.03 (−0.16; 0.09)	0.621	−0.001 (−0.11; 0.11)	0.981	0.34 (−2.74; 3.41)	0.825	−0.02 (−1.75; 1.71)	0.982	−0.02 (−0.76; 0.73)	0.965
Language score	−0.07 (−0.23; 0.89)	0.380	−0.04 (−0.18; 0.10)	0.569	0.66 (−3.06; 4.37)	0.719	−0.16 (−2.04; 2.08)	0.884	−0.40 (−1.28; 0.49)	0.364

*GA* Gestational age, *B* beta coefficient adjusted for, *GA* birth weight z-score, days of mechanical ventilation, age at MRI (for MRI data only), *CI* confidence interval, *ADC* Apparent diffusion coefficient.

Similarly, for the whole population the BSID-III language index correlated (Spearman) significantly with mean daily fluid intake (*p* = 0.002, r = 0.540), 28 days cumulative intake of calories (*p* = 0.007, r = 0.486) and carbohydrate (*p* < 0.001, r = 0.667), but only prior to covariate-adjustment (Table 4).

Additional regression analyses did not reveal any associations between nutrient intakes (or growth), and brain volumes (Supplementary Table S1), brain morphology (Supplementary Table S2), and neurodevelopment (Supplementary Table S3).

## Discussion

We investigated the impact of postnatal nutrition during the first four weeks on neurodevelopment, as evaluated by brain MRI at term and cognitive outcomes at 2 years, in a cohort of very preterm infants. We found no significant correlations between the intakes of the different macronutrients and growth, brain volumes, brain morphology, or cognition in a cohort where all macronutrient intakes were met according to current recommendations.

Our findings are in contrast to those of several previous studies that have reported a positive association between fat intake during the first two weeks of life and brain volumes at term,^19,20^ or between protein intake during the first four weeks and brain volumes at 30 weeks PMA.^21^ In the latter study this association was not sustained at term. A notable difference between these studies and the present is that the infants in those cohorts in general received a lower nutritional intake compared to those in our study cohort.

In support of our data Power et al.^3^ and Hansen-Pupp et al.^22^ found no association between protein and caloric intakes and brain volumes at term-equivalent age, in cohorts similar to ours regarding both gestational age and nutritional intakes.

Most interestingly, Tan et al.^23^ reported that an energy intake below 120 kcal/kg/day and a protein intake below 3 g/kg/d during the neonatal period was significantly associated with poorer neurodevelopment. Compared to our data their cohort on average received a lower caloric intake. Two other studies found that total protein intake during the first two and four weeks after birth, respectively, was the single nutritional factor positively correlated with cognitive scores (BSID-III) at 2 years.^1,24^ On the basis of these conflicting data, several authors have concluded that there is at present no consistent proof of early nutrition influencing neurodevelopmental outcomes.^3,25^

The challenges in all these studies are the obvious medical complexities of prematurity and the logistical challenges in providing optimal nutrition.^26^ Infants receiving “better” nutrition (higher cumulative intakes AND a greater proportion of enteral feeds) early after birth are typically healthier, more mature, and require less invasive interventions. Another consideration is that similar intakes may have varying impact on neurocognitive outcomes depending not only on the infant’s gestational age but also on when it occurs during the neonatal period.^27^ In spite of efforts to adjust for such confounding factors, it is still conceivable that these significantly influence the evaluated outcomes. Thus, it might require both a more homogenous preterm population and a much wider range of nutritional intakes to detect any effect.

The limitations of this study include the relatively small sample and its retrospective design, although we strongly believe our meticulous collection of nutritional data to be adequate. Furthermore, the targeted fortification approach may have contributed to the homogeneous nutritional intake within the cohort, potentially limiting the variability needed to detect any “dose-response effect” between nutrient intakes, and the outcomes evaluated. Other limitations include factors not accounted for in our analyses, such as infant medical condition, and the nutrition provided after the neonatal period, and beyond. The nutrition provided post-discharge, or any complicating illness indeed might individually influence growth and development, and blunt follow-up differences.

In conclusion, the results of this retrospective cohort study reveal no consistent associations between early postnatal nutritional intake in preterm infants and brain volumes at term or neurocognitive outcomes at 2 years. Given the population’s homogeneity in nutritional provision, we cannot preclude that larger deficits in nutritional intakes during the first month of life might have relevant negative effects on brain growth and later cognition.

## Supplementary information

Figure S1

Figure S2

Supplementary tables

## Data Availability

All relevant data for this study are included in the article with additional analyses available as supplementary material. Further enquiries can be directed to the corresponding author.

## References

[1] Coviello, C. et al. Effects of early nutrition and growth on brain volumes, white matter microstructure, and neurodevelopmental outcome in preterm newborns. *Pediatr. Res.* **83**, 102–110 (2018). 28915232 10.1038/pr.2017.227

[2] Kersbergen, K. J. et al. Relation between clinical risk factors, early cortical changes, and neurodevelopmental outcome in preterm infants. *Neuroimage* **142**, 301–310 (2016). 27395393 10.1016/j.neuroimage.2016.07.010

[3] Chan, S. H., Johnson, M. J., Leaf, A. A. & Vollmer, B. Nutrition and neurodevelopmental outcomes in preterm infants: a systematic review. *Acta Paediatr.* **105**, 587–599 (2016). 26813585 10.1111/apa.13344

[4] Isaacs, E. B. Neuroimaging, a new tool for investigating the effects of early diet on cognitive and brain development. *Front Hum. Neurosci.* **7**, 445 (2013). 23964224 10.3389/fnhum.2013.00445 PMC3734354

[5] Vasu, V. et al. Preterm nutritional intake and MRI phenotype at term age: a prospective observational study. *BMJ Open* **4**, e005390 (2014). 24860004 10.1136/bmjopen-2014-005390 PMC4039783

[6] Power, V. A. et al. Nutrition, Growth, Brain Volume, and Neurodevelopment in Very Preterm Children. *J. Pediatr.* **215**, 50–55.e3 (2019). 31561956 10.1016/j.jpeds.2019.08.031

[7] Gilbreath, D., Hagood, D. & Larson-Prior, L. A Systematic Review over the Effect of Early Infant Diet on Neurodevelopment: Insights from Neuroimaging. *Nutrients* **16**, 1703 (2024). 38892636 10.3390/nu16111703 PMC11174660

[8] Jobe, A. H. & Bancalari, E. Bronchopulmonary dysplasia. *Am. J. Respir. Crit. Care Med.* **163**, 1723–1729 (2001). 11401896 10.1164/ajrccm.163.7.2011060

[9] International Committee for the Classification of Retinopathy of Prematurity. The International Classification of Retinopathy of Prematurity revisited. *Arch. Ophthalmol.* **123**, 991–999 (2005). 16009843 10.1001/archopht.123.7.991

[10] Early Treatment For Retinopathy Of Prematurity Cooperative Group. Revised indications for the treatment of retinopathy of prematurity: results of the early treatment for retinopathy of prematurity randomized trial. *Arch. Ophthalmol.* **121**, 1684–1694 (2003). 14662586 10.1001/archopht.121.12.1684

[11] Rizzo, V., Capozza, M., Panza, R., Laforgia, N. & Baldassarre, M. E. Macronutrients and Micronutrients in Parenteral Nutrition for Preterm Newborns: A Narrative Review. *Nutrients* **14**, 1530 (2022). 35406142 10.3390/nu14071530 PMC9003381

[12] Schneider, J. et al. Evolution of T1 Relaxation, ADC, and Fractional Anisotropy during Early Brain Maturation: A Serial Imaging Study on Preterm Infants. *AJNR Am. J. Neuroradiol.* **37**, 155–162 (2016). 26494693 10.3174/ajnr.A4510 PMC7960197

[13] Arzoumanian, Y. et al. Diffusion tensor brain imaging findings at term-equivalent age may predict neurologic abnormalities in low birth weight preterm infants. *AJNR Am. J. Neuroradiol.* **24**, 1646–1653 (2003). 13679287 PMC7974006

[14] Papile, L. A., Burstein, J., Burstein, R. & Koffler, H. Incidence and evolution of subependymal and intraventricular hemorrhage: a study of infants with birth weights less than 1,500 gm. *J. Pediatr.* **92**, 529–534 (1978). 305471 10.1016/s0022-3476(78)80282-0

[15] Kidokoro, H., Neil, J. J. & Inder, T. E. New MR imaging assessment tool to define brain abnormalities in very preterm infants at term. *AJNR Am. J. Neuroradiol.* **34**, 2208–2214 (2013). 23620070 10.3174/ajnr.A3521 PMC4163698

[16] Beare, R. J. et al. Neonatal Brain Tissue Classification with Morphological Adaptation and Unified Segmentation. *Front. Neuroinform.* **10**, 12 (2016). 27065840 10.3389/fninf.2016.00012 PMC4809890

[17] Vaz, T. F. et al. Brain Extraction Methods in Neonatal Brain MRI and Their Effects on Intracranial Volumes. *Appl. Sci.* **14**, 1339 (2024).

[18] Johnson, S., Moore, T. & Marlow, N. Using the Bayley-III to assess neurodevelopmental delay: which cut-off should be used? *Pediatr. Res.* **75**, 670–674 (2014). 24492622 10.1038/pr.2014.10

[19] Schneider, J. et al. Nutrient Intake in the First Two Weeks of Life and Brain Growth in Preterm Neonates. *Pediatrics* **141**, e20172169 (2018). 29440285 10.1542/peds.2017-2169

[20] Ottolini, K. M. et al. Early Lipid Intake Improves Cerebellar Growth in Very Low-Birth-Weight Preterm Infants. *JPEN J. Parenter. Enter. Nutr.* **45**, 587–595 (2021). 10.1002/jpen.1868 PMC8496982 32384168

[21] van Beek, P. E. et al. Increase in Brain Volumes after Implementation of a Nutrition Regimen in Infants Born Extremely Preterm. *J. Pediatr.* **223**, 57–63.e5 (2020). 32389719 10.1016/j.jpeds.2020.04.063

[22] Hansen-Pupp, I. et al. Postnatal decrease in circulating insulin-like growth factor-I and low brain volumes in very preterm infants. *J. Clin. Endocrinol. Metab.* **96**, 1129–1135 (2011). 21289247 10.1210/jc.2010-2440

[23] Tan, M. J. & Cooke, R. W. Improving head growth in very preterm infants-a randomised controlled trial I: neonatal outcomes. *Arch. Dis. Child Fetal Neonatal Ed.* **93**, F337–F341 (2008). 18252814 10.1136/adc.2007.124230

[24] Cormack, B. E., Bloomfield, F. H., Dezoete, A. & Kuschel, C. A. Does more protein in the first week of life change outcomes for very low birthweight babies? *J. Paediatr. Child Health* **47**, 898–903 (2011). 21658149 10.1111/j.1440-1754.2011.02106.x

[25] Hansen-Pupp, I. et al. Circulatory insulin-like growth factor-I and brain volumes in relation to neurodevelopmental outcome in very preterm infants. *Pediatr. Res* **74**, 564–569 (2013). 23942554 10.1038/pr.2013.135

[26] Andrews, E. T., Ashton, J. J., Pearson, F., Beattie, R. M. & Johnson, M. J. Early postnatal growth failure in preterm infants is not inevitable. *Arch. Dis. Child Fetal Neonatal Ed.* **104**, F235–F241 (2019). 30135111 10.1136/archdischild-2018-315082

[27] Bloomfield, F. H., Jiang, Y., Harding, J. E., Crowther, C. A. & Cormack, B. E. ProVIDe Trial Group. Early Amino Acids in Extremely Preterm Infants and Neurodisability at 2 Years. *N. Engl. J. Med.* **387**, 1661–1672 (2022). 36322845 10.1056/NEJMoa2204886

