# Supplementary material for: Impact of early nutrition on brain development and neurocognitive outcomes in very preterm infants
Source: Pediatr Res. 2025 Mar 4;98(2):593–8. doi: 10.1038/s41390-025-03964-8 (PMC12454131; doi:10.1038/s41390-025-03964-8)
Supplement: Supplementary file 1 — Figure S1 [file 41390_2025_3964_MOESM1_ESM.pdf]

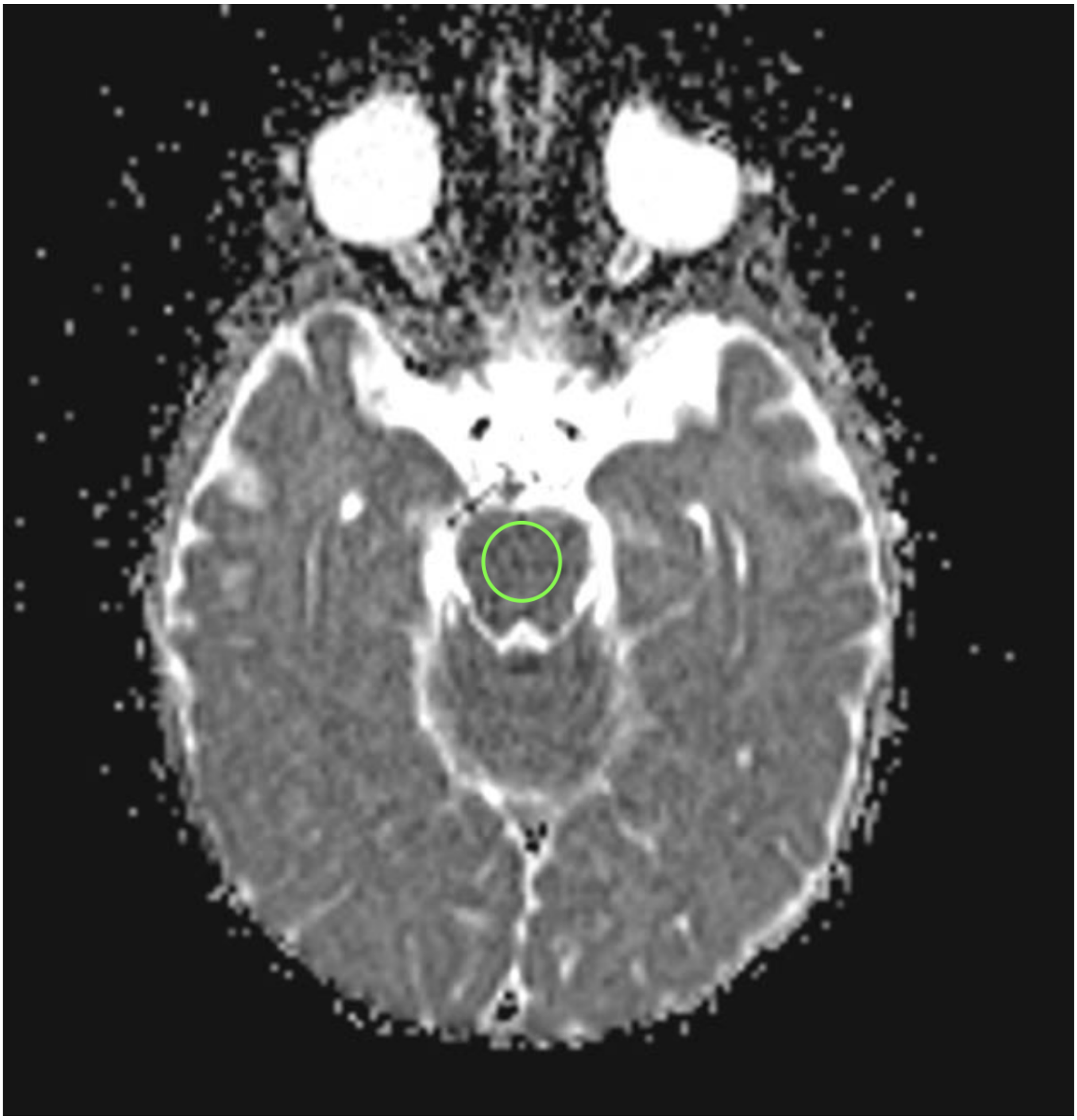

Figure S1. Representative sample of Apparent Diffusion Coefficients (ADC) derived from the Diffusion-Weighted Imaging (DWI; Single-shot EPI sequences, b-value = 0 and 800 s/mm^2) data measured in the pons, by outlining circle-shaped regions-of-interest with predefined radii (Schneider J, et al. Evolution of T1 Relaxation, ADC, and Fractional Anisotropy during Early Brain Maturation: A Serial Imaging Study on Preterm Infants. AJNR Am J Neuroradiol. 2016 Jan;37(1):155-62).
