# Supplementary material for: Impact of early nutrition on brain development and neurocognitive outcomes in very preterm infants
Source: Pediatr Res. 2025 Mar 4;98(2):593–8. doi: 10.1038/s41390-025-03964-8 (PMC12454131; doi:10.1038/s41390-025-03964-8)
Supplement: Supplementary file 2 — Figure S2 [file 41390_2025_3964_MOESM2_ESM.pdf]

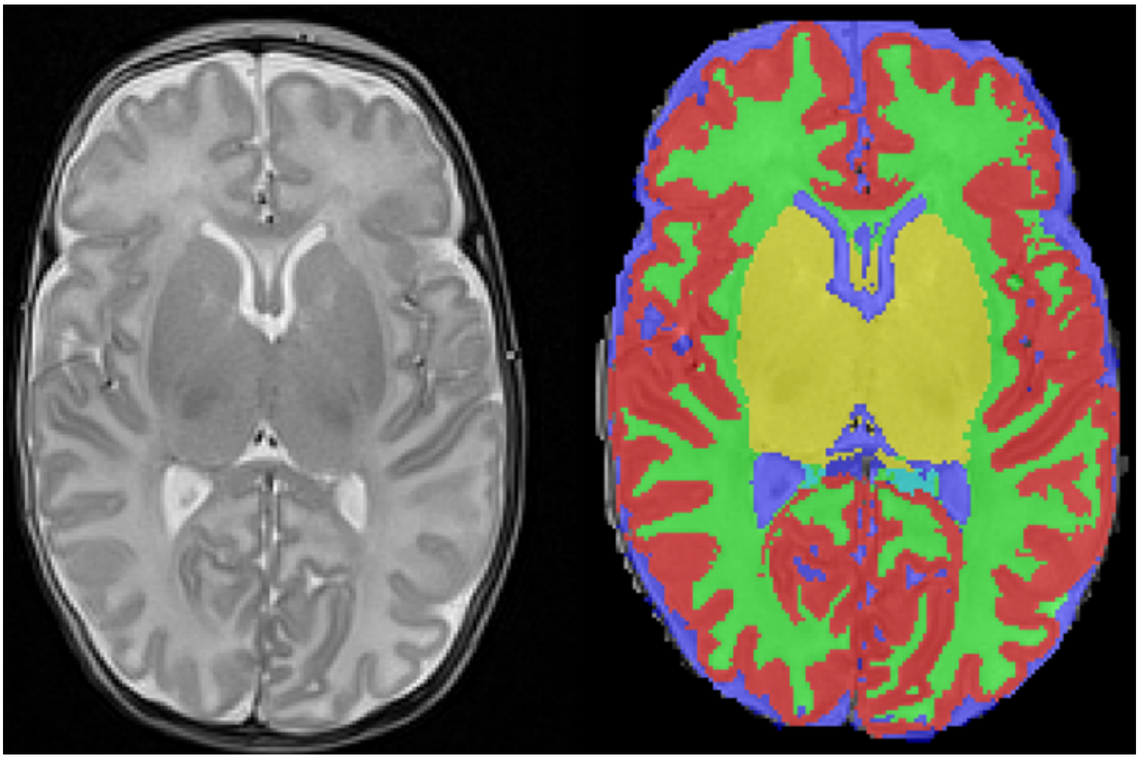

Figure S2. Representative sample of a brain T2W MRI axial slice (left), with tissue/structure segmentation (right) performed by MANTiS (Vaz TF et al. Brain Extraction Methods in Neonatal Brain MRI and Their Effects on Intracranial Volumes. *Applied Sciences*. 2024; 14(4):1339. https://doi.org/10.3390/app14041339). The overlay is color coded (cortical grey matter, red; white matter, green; cerebral spinal fluid, purple; deep nuclear grey matter, yellow; hippocampus, light blue).
