# Supplementary material for: Impact of early nutrition on brain development and neurocognitive outcomes in very preterm infants
Source: Pediatr Res. 2025 Mar 4;98(2):593–8. doi: 10.1038/s41390-025-03964-8 (PMC12454131; doi:10.1038/s41390-025-03964-8)
Supplement: Supplementary file 3 — Supplementary tables [file 41390_2025_3964_MOESM3_ESM.pdf]

Supplementary table 1. Multivariable linear regression of postnatal growth, cumulative nutritional intakes, and brain volumes

	Cerebellum	
	B (95 % CI)	p
Exceeded birth weight		
Day 7	0.30 (-2.00; 2.61)	0.793
Day 14	1.13 (-2.47; 4.73)	0.531
Proportion enteral feeds of total day 7	0.01 (-0.04; 0.05)	0.773
Enteral feeds only by day 14	2.19 (-0.03; 4.41)	0.053
Total fluid intake (mL/kg/d)		
Day 0-28	-0.01 (-0.03; 0.02)	0.588
Caloric intake (kcal/kg/d)		
Day 0-28	-0.001 (-0.02; 0.02)	0.962
Protein intake (g/kg/d)		
Day 0-28	-0.08 (-0.90; 0.75)	0.851
Fat intake (g/kg/d)		
Day 0-28	-0.01 (-0.34; 0.32)	0.963
Carbohydrates intake (g/kg/d)		
Day 0-28	-0.01 (-0.17; 0.15)	0.920

B, beta coefficient adjusted for: GA, birth weight z-score, days of mechanical ventilation, age at MRI; CI, confidence interval.

Supplementary table 2. Multivariable linear regression, and logistic regression as applicable, of postnatal growth, cumulative nutritional intakes, and MRI morphology

	IVH		PVL		VD	
	B (95 % CI)	p	B (95 % CI)	p	B (95 % CI)	p
Exceeded birth weight						
Day 7	0.58 (0.15; 1.01)	0.010	0.20 (-0.10; 0.51)	0.189	0.19 (-0.06; 0.44)	0.131
Day 14	0.13 (-0.67; 0.94)	0.741	0.13 (-0.43; 0.68)	0.648	-0.08 (-0.52; 0.36)	0.709
Proportion enteral feeds of total day 7	-0.01 (-0.02; 0.001)	0.074	-0.003 (-0.01; 0.002)	0.210	0.001 (-0.003; 0.01)	0.608
Enteral feeds only by day 14	-1.42 (-0.58; 0.29)	0.514	-0.46 (-0.73; -0.19)	0.001	-0.01 (-0.25; 0.23)	0.950
Total fluid intake (mL/kg/d)						
Day 0-28	-0.001 (-0.01; 0.004)	0.733	0.001 (-0.003; 0.004)	0.770	0.000 (-0.003; 0.003)	0.923
Caloric intake (kcal/kg/d)						
Day 0-28	-0.002 (-0.01; 0.003)	0.412	-0.001 (-0.004; 0.003)	0.735	0.001 (-0.002; 0.003)	0.608
Protein intake (g/kg/d)						
Day 0-28	-0.03 (-0.18; 0.12)	0.693	0.001 (-0.11; 0.11)	0.978	0.03 (-0.05; 0.12)	0.448
Fat intake (g/kg/d)						
Day 0-28	-0.04 (-0.11; 0.02)	0.179	0.003 (-0.05; 0.05)	0.897	0.01 (-0.03; 0.04)	0.715
Carbohydrates intake (g/kg/d)						
Day 0-28	-0.01 (-0.03; 0.04)	0.640	-0.01 (-0.03, 0.02)	0.511	0.004 (-0.02; 0.02)	0.680

B, beta coefficient adjusted for: GA, birth weight z-score, days of mechanical ventilation, age at MRI; CI, confidence interval; IVH, Intraventricular hemorrhage; PVL, Periventricular leukomalacia; VD, Ventricular dilatation.

Supplementary table 3. Multivariable linear regression of postnatal growth, cumulative nutritional intakes, and neurodevelopment

	Motor score	
	B (95 % CI)	p
Exceeded birth weight		
Day 7	4.93 (-3.91; 13.76)	0.266
Day 14	-0.65 (-16.19; 14.87)	0.932
Proportion enteral feeds of total day 7	0.09 (-0.07; 0.24)	0.259
Enteral feeds only by day 14	4.13 (-4.37; 12.62)	0.332
Total fluid intake (mL/kg/d)		
Day 0-28	0.02 (-0.08; 0.12)	0.655
Caloric intake (kcal/kg/d)		
Day 0-28	0.01 (-0.08; 0.09)	0.888
Protein intake (g/kg/d)		
Day 0-28	-0.66 (-2.82; 1.50)	0.536
Fat intake (g/kg/d)		
Day 0-28	0.04 (-1.20; 1.28)	0.947
Carbohydrates intake (g/kg/d)		
Day 0-28	0.10 (-0.49; 0.70)	0.724

B, beta coefficient adjusted for: GA, birth weight z-score, days of mechanical ventilation; CI, confidence interval.
